# SARS-CoV-2 Vaccination and Protection Against Clinical Disease: A Retrospective Study, Bouches-du-Rhône District, Southern France, 2021

**DOI:** 10.3389/fmicb.2021.796807

**Published:** 2022-01-18

**Authors:** Pierre-Edouard Fournier, Linda Houhamdi, Philippe Colson, Sébastien Cortaredona, Lea Delorme, Carole Cassagne, Jean-Christophe Lagier, Hervé Chaudet, Hervé Tissot-Dupont, Audrey Giraud-Gatineau, Florence Fenollar, Matthieu Million, Didier Raoult

**Affiliations:** ^1^IHU Méditerranée Infection, Marseille, France; ^2^Vecteurs —Infections Tropicales et Méditerranéennes (VITROME), Aix-Marseille Univ, Marseille, France; ^3^Microbes Evolution Phylogeny and Infections (MEPHI), Aix-Marseille Univ, Marseille, France; ^4^French Armed Forces Center for Epidemiology and Public Health (CESPA), Marseille, France

**Keywords:** COVID-19, SARS-CoV-2, vaccine, death, intensive care, hospitalization, protective effect

## Abstract

From January 18th to August 13th, 2021, 13,804 unvaccinated and 1,156 patients who had received at least one COVID-19 vaccine dose were tested qPCR-positive for SARS-CoV-2 in our center. Among vaccinated patients, 949, 205 and 2 had received a single, two or three vaccine doses, respectively. Most patients (80.3%) had received the Pfizer-BioNTech vaccine. The SARS-CoV-2 variants infecting vaccinated patients varied over time, reflecting those circulating in the Marseille area, with a predominance of the Marseille-4/20A.EU2 variant from weeks 3 to 6, of the Alpha/20I variant from weeks 7 to 25, and of the Delta/21A variant from week 26. SARS-CoV-2 infection was significantly more likely to occur in the first 13 days post-vaccine injection in those who received a single dose (48.9%) than two doses (27.4%, *p*< 10^–3^). Among 161 patients considered as fully vaccinated, i.e., >14 days after the completion of the vaccinal scheme (one dose for Johnson and Johnson and two doses for Pfizer/BioNTech, Moderna and Sputnik vaccines), 10 (6.2%) required hospitalization and four (2.5%) died. Risks of complications increased with age in a nonlinear pattern, with a first breakpoint at 54, 33, and 53 years for death, transfer to ICU, and hospitalization, respectively. Among patients infected by the Delta/21A or Alpha/20I variants, partial or complete vaccination exhibited a protective effect with a risk divided by 3.1 for mortality in patients ≥ 55 years, by 2.8 for ICU transfer in patients ≥ 34 years, and by 1.8 for hospitalization in patients ≥ 54 years. Compared to partial vaccination, complete vaccination provided an even stronger protective effect, confirming effectiveness to prevent severe forms of COVID-19.

## Introduction

Since the beginning of 2020, COVID-19 infected more than 254 million people and caused more than 5.1 million deaths worldwide (accessed on November, 23, 2021)^1^. Therefore, the development of vaccines raised hope to control the pandemic. However, due to the intensity of the pandemic and the overburden of healthcare systems in many countries, the use of new vaccines was authorized despite limited trials and safety data under the conditional marketing authorization rule (European Commission, 2006). Early clinical trials or mRNA vaccine for infectious diseases (rabies) were performed in 2013. Then, the first clinical trial of mRNA vaccine in lipid nanoparticles was performed in 2015 (influenza). However, before COVID-19, large scale use of mRNA vaccine in lipid nanoparticles and their safety in large-scale utilization in the real world were lacking (Dolgin, 2021). To date, several COVID-19 vaccines have been developed worldwide, mostly based on two technologies (mRNA and adenoviral vector) and 7.5 billion doses were administered (Ritchie et al., 2020). Among them, six have been used in priority, including the Pfizer-BioNTech (Ramasamy et al., 2021), the AstraZeneca (Walsh et al., 2020) and the Moderna (Baden et al., 2021) vaccines. The effectiveness of the Pfizer-BioNTech BNT162b2 and Moderna mRNA-1273 vaccines for preventing symptomatic infection was reported to be 60–80% > 14 days following a first dose and 90% with two doses (Amit et al., 2021; Hall et al., 2021a,b; Thompson et al., 2021a). Recent reports described increasing numbers of vaccine breakthrough infections (Bahl et al., 2021; Blanquart et al., 2021; Glatman-Freedman et al., 2021; Puranik et al., 2021; Scobie et al., 2021; Thompson et al., 2021b).

In our center, large-scale detection (Amrane et al., 2020) and treatment (Lagier et al., 2020; Million et al., 2021) of COVID-19 have been performed since January 2020. This enabled us to detect early SARS-CoV-2 breakthrough infections in vaccinated patients. Here we aimed at describing retrospectively the incidence and characteristics of SARS-CoV-2 vaccine breakthrough infections that were diagnosed in our institute, and their associations with related risks or predictors of hospitalization, admission to intensive care unit and death.

## Materials and Methods

### Study Design

The study is a retrospective cohort study of all SARS-CoV-2-positive patients diagnosed at Mediterranee Infection Institute from January 18th 2021, day of the first vaccine dose received by the first vaccinated patients who tested positive in our center, to August 13th 2021. We described the main characteristics of this reference population and compared the vaccinated and unvaccinated sub-populations, with stratification by age and infecting viral variant. Among vaccinated patients, we also compared those who were fully vaccinated (>14 days after the completion of the vaccinal scheme [one dose for Johnson & Johnson and two doses for Pfizer/BioNTech, Moderna and Sputnik vaccines]) to those who were only partially vaccinated (< 14 days after the first Johnson & Johnson dose or after the second Pfizer/BioNTech, Moderna and Sputnik vaccines).

### Study Area

Patients in this study lived in the Bouches-du-Rhône department, southern France.

### Population Under Study

SARS-CoV-2 infection was diagnosed using real-time reverse transcription (RT)-PCR (qPCR) on a nasopharyngeal swab. For each patient, we collected anonymously from the hospital database their date of birth, sex, symptoms when available, evolution, hospitalization in conventional medicine department or intensive care unit (ICU), death or recovery. “Vaccinated” patients referred to all patients who had received at least one vaccine dose; “fully vaccinated” patients referred to those who had a complete vaccine scheme; and “partially vaccinated” patients referred to those who had received an incomplete vaccine scheme.

### Methods

SARS-CoV-2 qPCR from nasopharygeal swabs was performed as previously described (Fournier et al., 2020). Genotypes were determined using genome next-generation sequencing and analysis, as previously described (Colson et al., 2021).

### Statistical Analysis

Qualitative variables (sex, admission to hospital or ICU, death) of vaccinated and unvaccinated patients in our cohort were compared in univariate analyses using the Fisher’s exact test, and the Student’s *t*-test was used to compare means of quantitative variables [age, delay between vaccine administration and SARS-CoV-2 detection by qPCR, cycle threshold (Ct) value]. To unequivocally test the efficacy of COVID-19 vaccination, symptoms were not taken into account in the statistical analysis as this information was missing for many patients and largely depended on physicians’ subjectivity.

In addition, age being identified as a major prognostic factor in COVID (Lagier et al., 2020), to determine whether the increase in risk was linear with age, we used a ROC analysis for age on death, ICU admission and hospitalization. Change points in the age related predictive values were determined by finding the multiple structural changes of the series’ linear model estimated by least-squares, using the method introduced by Bai and Perron (2003). We systematically considered the first change point of the optimal partition of the series’ ascending part for detecting the significative predictive value acceleration. Moreover, it was reported that the evolution was significantly different according to sex (male sex associated with a worse prognosis) (Lagier et al., 2021), and viral variants (Hoang et al., 2021). The associations between outcomes (death, ICU and hospitalization) and vaccination (at least 1 dose of vaccine) were assessed using multivariate logistic models with and without stratification. Models were adjusted on the following variables: sex, age, variant and partial or complete vaccination. Because the number of patients with at least 1 dose of vaccine was very low for the Marseille-4/20A.EU2 variant (*n* = 36), models were performed on the subgroup of patients with the Alpha/20I or Delta/21A variant (*n* = 11,624).

Multivariate analysis is critical to test if vaccination is a predictor independent of other covariates (age, sex, and variants), however, this global approach does not allow to detect a subgroup with an aberrant or discordant effect. Stratified analyses allow to assess whether observed effects are reproducible and consistent across subgroups according to age groups (≥ or < age determined by breakpoint Bai-Perron testing), sex and variant. Models were adjusted on the two remaining adjustment variables (e.g., adjusted on sex and variant when stratified on age). Adjusted odds ratios of the vaccination variable (at least one dose) estimated in each model were reported on forest plots. This approach allowed us to check whether the observed effect of vaccination was consistent in the whole population and across different clinically relevant subgroups.

Finally, we compared the efficiency of a complete to an incomplete vaccination scheme (1 or 2 doses, depending on the vaccine) using logistic regression models on the population infected with an Alpha/20I or Delta/21A variant (*n* = 11,624) with adjustment for age, sex and variant. Goodness of fit of the multivariate logistic regressions was assessed using the Hosmer-Lemeshow (HL) test. A two-sided α value of less than 0.05 was considered to be statistically significant. Analyses were carried out using SAS 9.4 statistical software (SAS Institute, Cary, NC, United States).

## Results

### Population Under Study

From January 18st, to August 13th, 2021, we performed 299,561 SARS-CoV-2 qPCR assays on nasopharyngeal specimens from 180,812 patients. Of these, qPCR was positive in 14,960 patients (Supplementary Table 1). This reference population included 7,196 men [mean age 43 (0–100) years] and 7,764 women [mean age 44 (1–103) years]. Among these patients, 1,156 were vaccinated against SARS-CoV-2, including 161 and 995 who were fully and partially vaccinated, respectively. Vaccinated patients included 551 men [mean age 52 (15–96) years] and 605 women [49 (14–103) years]. Nine hundred and fourty-nine patients had received a single vaccine dose whereas 205 and 2 had received two and three doses, respectively. Among the 965 patients for whom this information was available, 775 (80.3%) had received the Pfizer-BioNTech vaccine [including 587 (60.8%), 186 (19.2%), and 2 (0.2%) who had received one, two and three doses, respectively], 107 (11.1%) had received the AstraZeneca vaccine (one and two doses in 100 and seven patients, respectively), 62 (6.4%) the Moderna vaccine (one and two doses in 56 and six patients, respectively), 17 (1.8%) the Johnson & Johnson vaccine (single dose), two (0.2%) the SpoutnikV vaccine (two doses) and two (0.2%) a first dose of AstraZeneca and a second dose of Pfizer-BioNTech. The vaccine type was not available for 191 patients (Table 1 and Supplementary Table 2).

**TABLE 1:** Univariate comparison of fully vaccinated, partially vaccinated and unvaccinated patients (*n* = 14,960).

	Number	Sex ratio (M/F)	Mean age ± SD	Median age	Symptomatic (%)	Hospitalization (%)	Admission to ICU (%)	Death (%)	Mean Ct value ± SD
Fully vaccinated	161	77/84	56.7 ± 22.3*	57	139 (86.3)	10 (6.2)	0 (0.0)	4 (2.5)	21.8 ± 5.4
Partially vaccinated	995	474/521	49.1 ±19.1*	51	815 (81.9)	60 (6.0)	9 (0.9)	11 (1.1)	22.5 ± 5.3
Unvaccinated patients	13,804	6,645/7,159	42.9 ± 19.1*	42	11,185 (81.0)	980 (7.1)	229 (1.7)	269 (2.0)	22.4 ± 5.2

*Comparisons of fully vaccinated, partially vaccinated and unvaccinated patients’ ages were statistically different (p < 10^–3^).

The detail of the viral variants infecting these patients is presented in Supplementary Table 3. Patients were mostly infected with the Alpha/20I variant, regardless the vaccine and number of doses used (Supplementary Table 4). However, the most frequent variant varied over time, the Marseille-4/20A.EU2 variant being predominant from weeks 3 to 6, the Alpha/20I variant being predominant from weeks 7 to 25, and the Delta/21A variant becoming majority from week 26 (Supplementary Figure 1). This evolution strictly paralleled the temporal distribution of variants among all infected patients, whatever their vaccination status, with the exception of week 20, during which we investigated a cluster of Delta/21A variant infections in a nursing home, which explains the peak of weeks 20–21 in Supplementary Figure 1.

**FIGURE 1:**
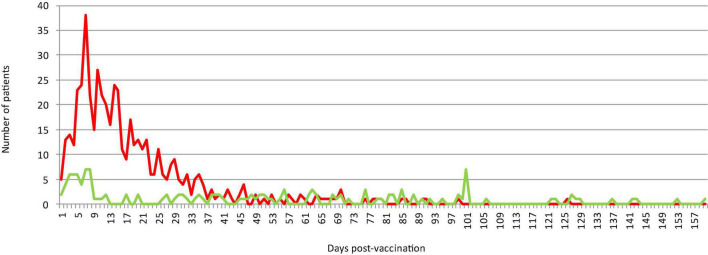
Temporal distribution of the numbers of patients who experienced SARS-CoV-2 infection following vaccination, according to the delay between vaccination and infection diagnosis. The red and green curves indicate patients who received a first or second dose, respectively.

The mean delays between vaccine administration and SARS-CoV-2 detection by qPCR were 18.9 ± 18.4 (range 1–145), 56.9 ± 46.0 (range 1–171) and 38 (21 and 55) days for patients who had received one, two, and three doses, respectively (Supplementary Table 5 and Figure 1). SARS-CoV-2 infection was significantly more likely to occur in the first 13 days post-vaccine injection in those who received a single dose [50% (310 out of 620)] than two doses [27.4% (56 out of 204), *p*< 10^–3^]. Regarding the viral load, the mean cycle threshold (Ct) value obtained for the qPCR-positive specimen at diagnosis in patients after one, two or three vaccine doses was not statistically different, with 22.4 ± 5.3 (range, 11–32) (available for 847 patients), 22.4 ± 5.1 (5–34) (available for 183 patients) and 20.9 ± 4.5 (16.3, 25.6), respectively.

### Statistical Analysis

Fully (*n* = 161) and partially (*n* = 995) vaccinated patients were significantly older than those who were not vaccinated (*n* = 13,804, *p*< 10^–3^ and *p*< 10^–3^, respectively) (Table 1). Their mean Ct value at diagnosis was 22.4 ± 5.3. A majority of fully vaccinated patients (86.3%) were symptomatic, but only 10 (6.2%) required hospitalization (none in ICU) and 4 (2.5%) died (Table 1). When compared to partially vaccinated and unvaccinated patients, fully vaccinated patients did not differ significantly in terms of mean Ct value at diagnosis (*p* = 0.735 and 0.228, respectively), admission to hospital (*p* = 0.860 and 0.759, respectively), admission to ICU (*p* = 0.621 and 0.118, respectively), and death (*p* = 0.144 and 0.560, respectively). However, although not statistically different, we observed lower Ct values and higher rates of death in fully vaccinated than unvaccinated patients, whereas the latter were more likely to be admitted to ICU. When stratified by viral variant, significant differences in age between fully and unvaccinated patients were also noted (Supplementary Table 6), vaccinated patients being older. In addition, fully vaccinated patients infected with the Alpha/20I variant were more likely to be male (*p* = 0.026 and 0.046, respectively) than partially and unvaccinated patients. Apart from an older age, partially vaccinated patients infected with the Beta/20H variant did not differ statistically from unvaccinated individuals (Supplementary Table 6). Among patients infected with the Eta/21D or Marseille-4/20A.EU2 variants, no significant difference in terms of outcome and hospitalization was observed among patients with regard to their vaccination status. In contrast, the group of unvaccinated patients infected with the remaining variants exhibited a significantly higher rate of ICU requirement (*p* = 0.04).

When comparing the characteristics of infections caused by the main three variants (Alpha/20I, Delta/21A and Marseille-4/20A.EU2, Supplementary Table 6), we observed that vaccinated patients infected with the Alpha/20I and Marseille-4/20A.EU2 variants did not differ significantly. However, vaccinated patients infected with the Delta/21A variant were less likely to be admitted to hospital (1.1 vs. 10.1%, *p <* 10^–3^) and ICU (0.0 vs. 1.4%, *p* = 0.005) than those infected with the Alpha/20I variant. Unvaccinated patients infected with the Marseille-4/20A.EU2 were less likely to be male (45.2 vs. 48.5 and 48.9%, *p* = 0.004 and 0.006, respectively) but more likely to be hospitalized (9.6 vs. 7.4 and 4.0%, *p*< 10^–3^ and < 10^–3^, respectively) and die (3.6% vs. 1.7 and 0.7%, *p*< 10^–3^ and < 10^–3^, respectively) than unvaccinated patients infected with the Alpha/20I and Delta/21A variants, respectively. In addition, unvaccinated patients infected with the Delta/21A variant were less likely to be admitted to hospital (4.0 vs. 7.4%, *p <*10^–3^) and ICU (0.9 vs. 1.7%, *p* = 0.001), and to die (0.7 vs. 1.7%, *p<*10^–3^) than those infected with the Alpha/20I variant.

**FIGURE 2:**
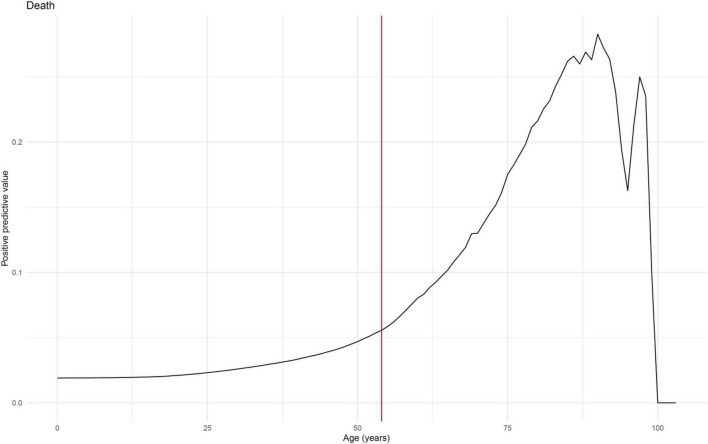
Predictive value of age on death (*n* = 14,960). Predictive value (probability of the event for ≥ age) is shown for death. Change points in the age related predictive values was determined by finding the multiple structural changes of the series’ linear model estimated by least-squares, using the method introduced by Bai and Perron (2003). We considered the first change point of the optimal partition of the series’ ascending part for detecting the significative predictive value acceleration. Optimal partitions of the ascending phases correspond to an acceleration at age 54.

To clarify factors predictive of the natural history of COVID-19 and the role of vaccination (at least 1 dose of vaccine), we first analyzed the role of age on the entire cohort (*n* = 14,960). We found that the increase in risk was not linear for the three considered outcomes (death, admission to ICU and hospitalization). Change point analyses of the age related predictive values showed that the optimal partitions of the ascending phases correspond to an acceleration at ages 54, 53, and 33 for the death, hospitalization and ICU transfer risks, respectively (Figure 2 and Supplementary Figures 2, 3) and these values were used as thresholds for age-stratified analysis. Then, the confounding role of age, sex and variant were controlled for by multivariate and stratification models.

To clarify the potential role of vaccination status on outcome among patients with either of the three main variants in our series, i.e., Alpha/20I, Delta/21A, or Marseille-4/20A.EU2, we first observed the proportion of patients who died (Table 2), were transferred to ICU (Supplementary Table 7) or were hospitalized (Supplementary Table 8) after stratification by age. For the Marseille-4/20A.EU2 variant, only 36 patients had at least 1 dose of vaccine, limiting any conclusions for these patients. For the Alpha/20I variant, 7,894 patients could be included, of whom 494 had received at least 1 dose of vaccine (6.3%). For the Delta/21A variant, 3,730 patients were included of whom 564 were vaccinated (15.1%).

**TABLE 2:** Fatality rate according to SARS-CoV-2 variant, age and vaccinal status (*n* = 14,114*).

	Marseille-4/20A.EU2 variant (*n* = 2,490)					
	Unvaccinated			Vaccinated (at least one dose)		
	n Total	n Deaths	Fatality rate (%)	n Total	n Deaths	Fatality rate (%)
**Age**						
<55	1,527	6	0.4	12	0	0.0
≥ 55	927	83	9.0	24	2	8.3
Total	2,454	89	3.6	36	2	5.6

	**Alpha/20I variant (*n* = 7,894)**					

**Age**						
<55	5,206	14	0.3	173	0	0.0
≥ 55	2,194	110	5.0	321	7	2.2
Total	7,400	124	1.7	494	7	1.4

	**Delta/21A variant (*n* = 3,730)**					

**Age**						
<55	2,585	1	0.0	410	0	0.0
≥ 55	581	22	3.8	154	3	1.9
Total	3,166	23	0.7	564	3	0.5

*Only patients with the Alpha/20I, Delta/21A or Marseille-4/20A.EU2 variants are included.

**FIGURE 3:**
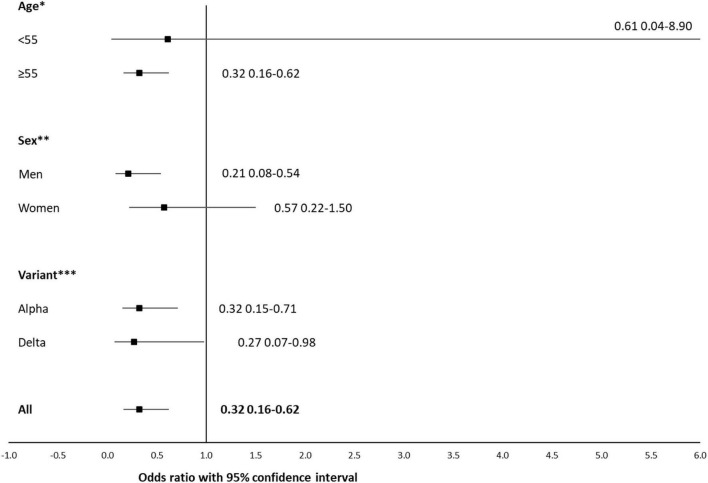
Forest plot showing the influence of age, sex and variant on association between death and vaccination (partially or fully vaccinated). Stratified multivariate logistic regression (*n* = 11,624*). Odds ratios and 95% confidence intervals are indicated for all comparisons. Only patients with the Alpha/20I or Delta/21A variants are included. *Multivariate model is stratified on age and adjusted on sex and variant. **Multivariate model is stratified on sex and adjusted on age and variant. ***Multivariate model is stratified on variant and adjusted on age and sex.

When the effect of vaccination (at least one dose) was assessed for mortality in the 11,624 patients with an Alpha/20I or Delta/21A variant with adjustment for age, sex, and variant, a significant protective effect was observed among patients with at least one dose with a factor of 3.1 [adjusted OR (aOR) 95% CI 0.32, 0.16–0.62, Table 3]. This protective effect was observed for all subgroups (Figure 3) but was significant only for individuals aged 55 years and older (aOR 95% CI 0.32, 0.16–0.62), male gender (aOR 95% CI 0.21 0.08–0.54) and the Alpha/20I (aOR 95% CI 0.32 0.15–0.71) and Delta/21A (aOR 95% CI 0.27, 0.07–0.98) variants. Among those under 55 years, for any of the Alpha/20I, Delta/21A and Marseille-4/20A.EU2 variants, no death was observed among the vaccinated. In unvaccinated patients under 55 years of age, there was one death in patients infected with the Delta/21A variant (Table 2 and Figure 3).

**TABLE 3:** Multivariate logistic regression analyzing the fatality rate (a), the rate of admission to ICU (b) and the rate of hospitalization (c) according to SARS-CoV-2 variant, sex, age and vaccinal status.

Death**					

Effect	Number without outcome	Number with outcome	Adjusted odds ratio with 95% CI		
**Sex**					
Women (ref.)*	5,922	54			
Men	5,545	103	2.64	1.85	3.77
**Age**			1.11	1.10	1.13
**Variant**					
Delta (ref.)*	3,704	26			
Alpha/20I	7,763	131	1.57	0.99	2.46
Vaccination status*****					
Unvaccinated (ref.)*	10,419	147			
Partially vaccinated	899	7	0.32	0.15	0.69
Fully vaccinated	149	3	0.32	0.09	1.09
*Partially or fully vaccinated*	1,048	10	0.32	0.16	0.62

Admission to ICU***					

**Sex**					
Women (ref.)*	5,927	49			
Men	5,533	115	2.66	1.90	3.73
**Age**			1.05	1.04	1.06
**Variant**					
Delta (ref.)*	3,702	28			
Alpha/20I	7,758	136	1.56	1.04	2.35
Vaccination status*****					
Unvaccinated (ref.)*	10,409	157			
Partially vaccinated	899	7	0.41	0.19	0.86
Fully vaccinated	152	0	0.11	0.01	1.88
*Partially or fully vaccinated*	1,051	7	0.36	0.17	0.73

Hospitalization****					

**Sex**					
Women (ref.)*	5,645	331			
Men	5,250	398	1.40	1.20	1.64
**Age**			1.06	1.06	1.07
**Variant**					
Delta (ref.)*	3,598	132			
Alpha/20I	7,297	597	1.57	1.28	1.92
Vaccination status*****					
Unvaccinated (ref.)*	9,893	673			
Partially vaccinated	857	49	0.58	0.43	0.80
Fully vaccinated	145	7	0.31	0.14	0.68
*Partially or fully vaccinated*	1,002	56	0.53	0.40	0.72

Only patients with the Alpha/20I and Delta/21A variants are included (n = 11,624*). *Ref. = Reference patient group. **Hosmer-Lemeshow (HL) test: p = 0.062. By comparing complete to partial vaccination, the differences were not significant (p = 0.987). ***Hosmer-Lemeshow (HL) test: p = 0.243. By comparing complete to partial vaccination, the differences were not significant (p = 0.387). ****Hosmer-Lemeshow (HL) test: p = 0.4147. By comparing complete to partial vaccination, the differences were not significant (p = 0.135). *****Vaccination status was tested as a binary variable (unvaccinated/partially or fully vaccinated) and a 3-level variable (unvaccinated/partially/fully vaccinated).

For ICU admission (Supplementary Table 7 and Supplementary Figure 4), in those 34 years and older, having received at least 1 dose of vaccine was associated with a significant benefit of a factor of 2.8 (aOR 95% CI 0.36, 0.18–0.74), but this effect was not significant for those younger than 34 years (aOR 95% CI 0.85, 0.05–13.63). For hospitalization (Supplementary Table 8 and Supplementary Figure 5), in those over 54 years of age, the protective effect of the vaccine (at least one dose) was significant (reduction by a factor of 1.9, aOR 95% CI 0.53, 0.40–0.72). This protective effect was also seen in the under-54 age group with a relatively similar effect size (reduction by a factor of 2.3, aOR 95% CI 0.44, 0.20–0.94).

Finally, when comparing the efficiency of a complete to an incomplete vaccination scheme on the population infected with an Alpha/20I or Delta/21A variant with adjustment for age, sex and variant, the statistical power was reduced by further stratification of the vaccinated population (*n* = 1058 or 9.1% of our total Alpha/20I or Delta/21A population, Table 3) into partially vaccinated (*n* = 906, 7.8%) and fully vaccinated (*n* = 152, 1.3%). Nevertheless, a complete vaccination scheme divided by 3.1 the risk of death (aOR 0.32, 95% CI 0.09–1.09). Strikingly, this was not different from the protective effect observed for partial vaccination (aOR 0.32, 0.15–0.69, *p* = 0.987). In contrast, the risk of transfer to ICU was divided by 9.1 in case of complete vaccination (aOR 0.11, 0.01–1.88) vs. 2.4 with a partial regimen (aOR 0.41, 0.19–0.86), with no significant difference (*p* = 0.387). The risk of hospitalization was divided by 3.2 (aOR 0.31, 0.14–0.68) for a complete regimen vs. 1.7 for a partial regimen (aOR 0.58, 0.43–0.80), without significant difference (*p* = 0.135).

Overall, the benefit of partial or complete vaccination was higher for death (risk divided by a factor of 3.1) than ICU transfer (2.8) and hospitalization (1.8). Complete vaccination exhibited a better effect than partial vaccination for ICU transfer (9.1 vs. 2.4) and hospitalization (3.2 vs. 1.7) but not for death (3.1 both for partial and complete vaccination) (Table 3).

## Discussion

We herein reported 1,156 patients who experienced SARS-CoV-2 infection despite having received a single or two doses of COVID-19 vaccine. While vaccination was unable to prevent infection in 1,156 patients reported here, it was associated with a significant reduction of the risk of death, ICU transfer and hospitalization, independently from age, sex and infecting viral variant.

Vaccine efficacy, although superior to 90% in phase III studies for mRNA vaccines, does not reach 100% (Glatman-Freedman et al., 2021; Puranik et al., 2021). Thus, vaccine breakthrough infection is expected to occur. In their report from January 1st to April 30th, 2021, the CDC reported 10,262 cases of vaccine breakthrough infections with a hospitalization rate of 10% and a lethality rate of 2% (CDC Covid-19 Vaccine Breakthrough Case Investigations Team, 2021). In our case series, the hospitalization and fatality rates were in the same range, being 6.0 and 0.9%, respectively. However, the severity of COVID-19 appeared to decrease over time in vaccinated patients in our series, with decreasing hospitalization and mortality rates from the Marseille-4/20A.EU2 to the Alpha/20I, Delta/21A, Beta/20H, and Eta/21D variants (Supplementary Table 3). Whether these differences result from an increasing degree of vaccine protection and/or from a progressive decrease in pathogenicity among variants, as previously described (Dao et al., 2021), is as-yet unknown.

We observed that in patients who had received a single vaccine dose, 50% of the first qPCR-positive results were obtained during the first 13 days. This result is in accordance with several studies. Keehner et al. (2021) reported 379 asymptomatic cases of SARS-CoV-2 infections among vaccinated health care workers from the University of California in San Diego and Los Angeles, accounting for an incidence of 1.03%. Among the 342 individuals who had received a first dose, the infection was detected within the first 2 weeks in 79% of cases. In addition, Bernal et al. (2021) reported that 39% of 1,537 patients older than 80 years who tested SARS-CoV-2-positive after vaccination by the Pfizer-BioNTech vaccine after the first dose but before the second dose were diagnosed during the 9 days post-injection. Moreover, this higher risk of being tested positive for SARS-CoV-2 early after vaccination was also reported in a large Scottish population by Vasileiou et al. (2021) who reported a significantly higher rate of infections in the first 2 weeks following the first dose. However, in contrast with the study from Keehner et al. (2021) in which 81% of infections following the second dose were detected within the first 2 weeks, we observed that infections following the second dose occurred mostly later than the 15th day (72.5%).

In our cohort, we also observed that, although not significantly, fully vaccinated patients exhibited a higher death rate than unvaccinated individuals. We assume that this results from a significantly older age of the former population (*p*< 10^–3^). As a matter of fact, we were able to demonstrate an increasing risk of COVID-19 complications with age that was not linear but accelerated from the age of 55 (Figure 2), which is younger than the age usually considered to define the population at risk (> 65 years) (Levin et al., 2020). In patients 55-years old and older, vaccination exhibited a protective effect against mortality caused by the Delta/21A or Alpha/20I variants, with a risk divided by 3.1 (Tables 2, 3), but no effect was observed on mortality in the under-55 s, due to the low mortality in this age group. A very substantial effect was observed for admission to ICU, with a risk divided by 2.8 in the over-55 s and by 3.6 in the under-55 s, but significant only in the over-55 s (Supplementary Figure 4). Finally, for hospitalization, a substantial and significant protective effect was observed, regardless of age and vaccination scheme (partial or complete), in patients infected with the Alpha/20I and Delta/21A variants (Table 3 and Supplementary Figure 5). When considering the protective effect of complete vs. partial vaccination, our analysis suggested that fully vaccinated patients had a lower risk of admission to ICU and hospitalization, but not of death, than those who were partially vaccinated (Table 3), although these differences were not significant due to a too small number of fully vaccinated patients. Similar findings were published by Bahl et al. (2021) who demonstrated in Michigan that fully vaccinated patients were less likely, although not statistically, to develop severe complications than partially vaccinated individuals. In a large study of more than 615,000 Delta/21A variant infections in 13 U.S. jurisdictions, Scobie et al. (2021) also observed that full vaccination reduced significantly the risk of hospitalization and death, in particular in patients older than 65-yo.

We acknowledge the fact that our study suffered from several drawbacks. First, our study design only enabled us to evaluate one of the three levels of effects from vaccines, that is the protection against clinical disease. The other two levels of effects (protection against infection and protection against transmission) could not be evaluated. Second, we observed a high number of symptomatic infections in vaccinated patients. This is consistent with a recent report of our team in which 89% of vaccinated infected patients were symptomatic, as compared to 80% of unvaccinated infected patients (Boschi et al., 2021). This most likely results from the fact that these patients are more likely to seek medical care and be tested for COVID-19 than asymptomatic individuals. Thus, it is likely that our results underestimate the proportion of asymptomatic vaccine breakthrough infections. Third, we cannot exclude a bias in the selection of patients tested in our institute, originating mainly from southern France.

Therefore, according to our results, the currently available vaccines do not prevent all SARS-CoV-2 infections. However, we observed a reduction in the risk of death by a factor of 3.1 in the over-55s, a reduction in the risk of admission to ICU by a factor of 2.7 regardless of age, and a reduction in the risk of hospitalization by a factor of 1.8 regardless of age. These protective effects of vaccination are very large and validate the place of vaccination in the arsenal of response to COVID-19.

## Data Availability Statement

The original contributions presented in the study are included in the article/Supplementary Material, further inquiries can be directed to the corresponding author/s.

## Ethics Statement

The study was approved by the ethical committee of the Mediterranee Infection institute under reference 2021-023. Access to the patients’ biological and registry data issued from the hospital information system was approved by the data protection committee of Assistance Publique-Hopitaux de Marseille (APHM) and was recorded in the European General Data Protection Regulation registry under number RGPD/APHM 2019-73.

## Author Contributions

P-EF and DR: conceptualization. P-EF, LH, PC, SC, LD, HC, and MM: methodology. P-EF, LH, PC, SC, LD, CC, J-CL, HC, HT-D, AG-G, FF, MM, and DR: data and investigation. P-EF, LH, PC, SC, LD, CC, MM, and DR: original draft preparation. P-EF: review and editing of the manuscript. All authors: supervision. All authors have read and agreed to the final version of the manuscript.

## Conflict of Interest

The authors declare that the research was conducted in the absence of any commercial or financial relationships that could be construed as a potential conflict of interest.

## Publisher’s Note

All claims expressed in this article are solely those of the authors and do not necessarily represent those of their affiliated organizations, or those of the publisher, the editors and the reviewers. Any product that may be evaluated in this article, or claim that may be made by its manufacturer, is not guaranteed or endorsed by the publisher.

## Footnotes

1
https://www.ecdc.europa.eu/en/geographical-distribution-2019-ncov-cases
