# Supplementary figures and images for: SARS-CoV-2 Vaccination and Protection Against Clinical Disease: A Retrospective Study, Bouches-du-Rhône District, Southern France, 2021
Source: Front Microbiol. 2022 Jan 18;12:796807. doi: 10.3389/fmicb.2021.796807 (PMC8803903; doi:10.3389/fmicb.2021.796807)

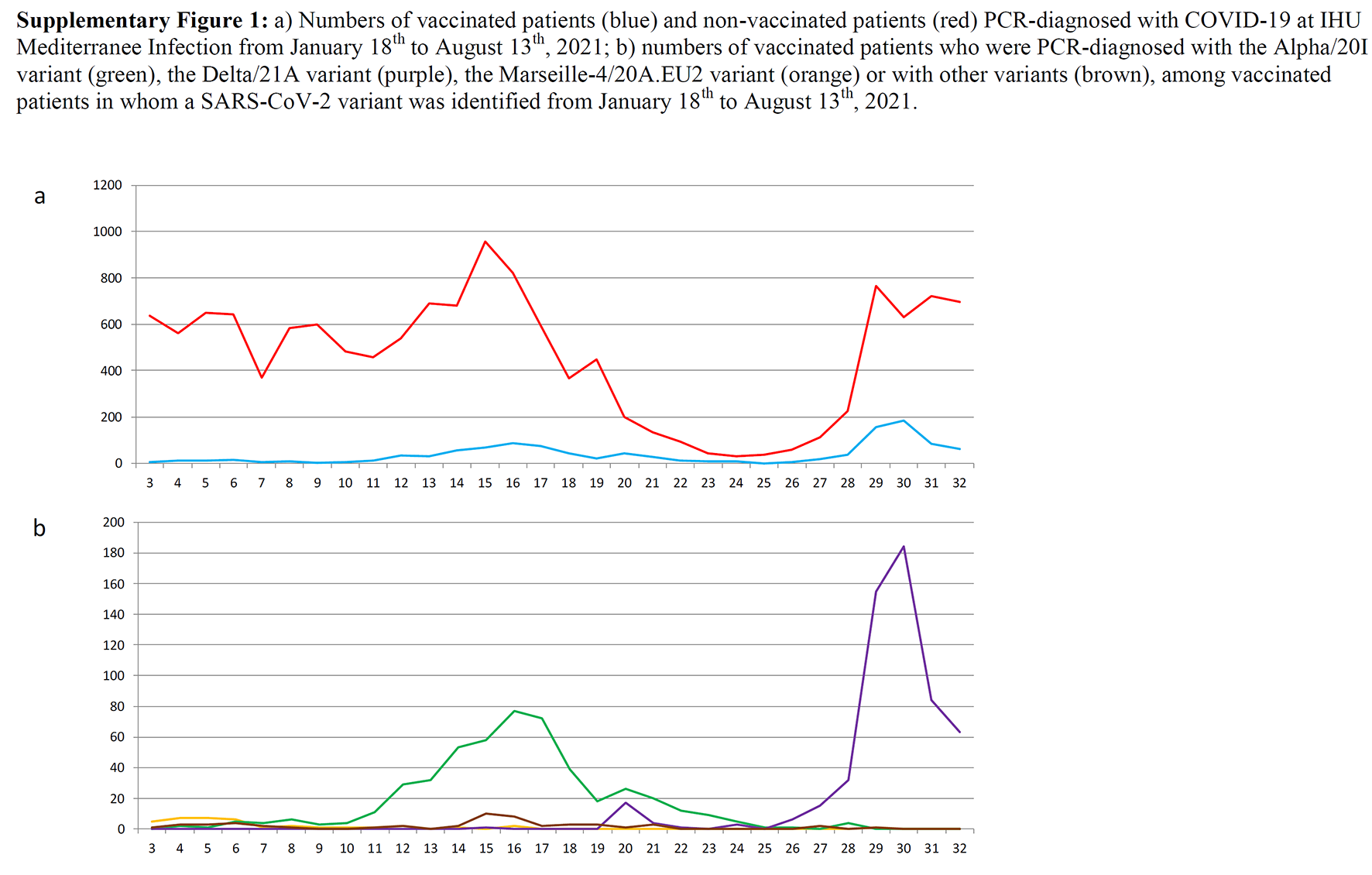

Supplement: Supplementary file 1 [file Image_1.tif]

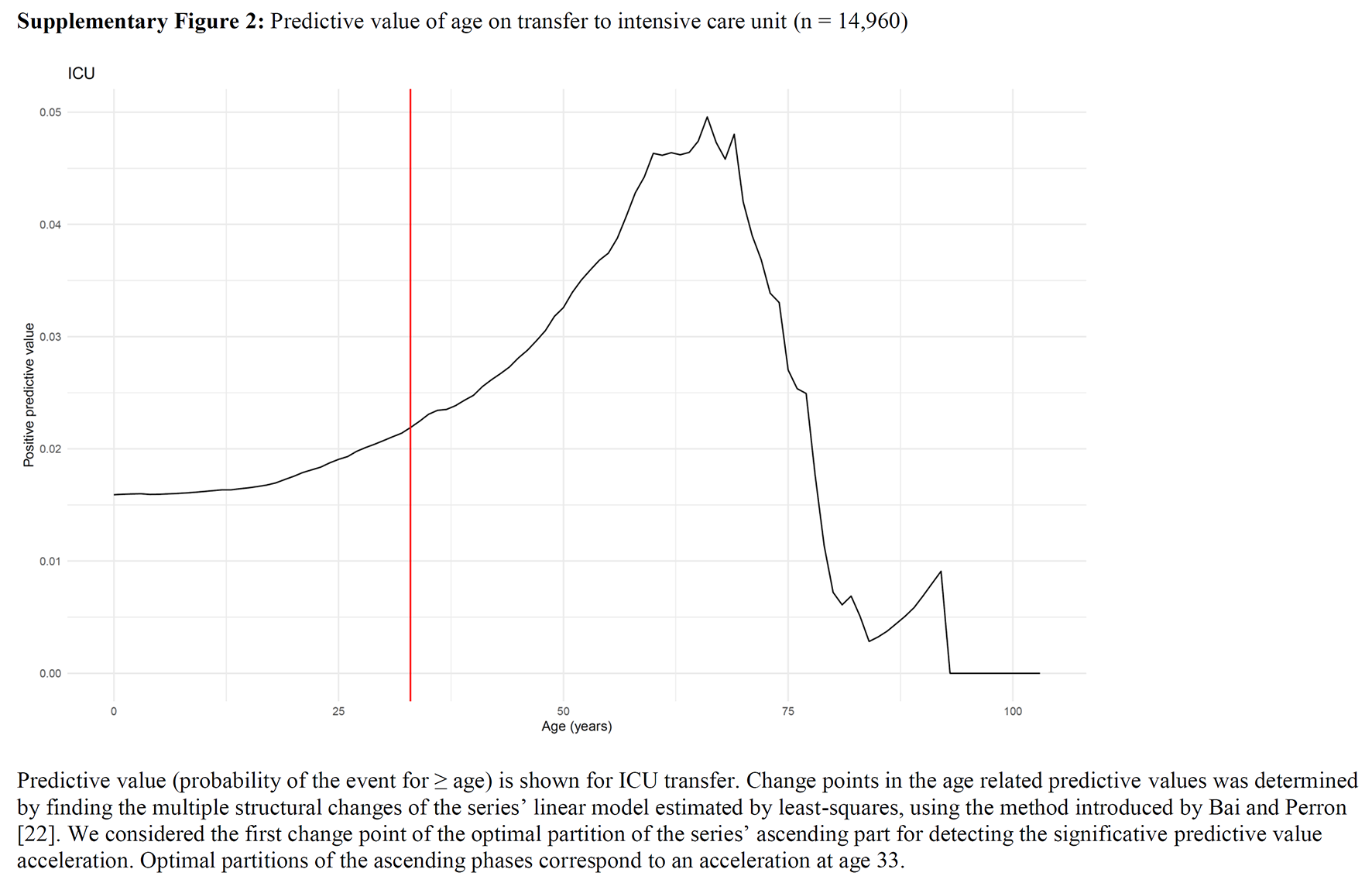

Supplement: Supplementary file 2 [file Image_2.tif]

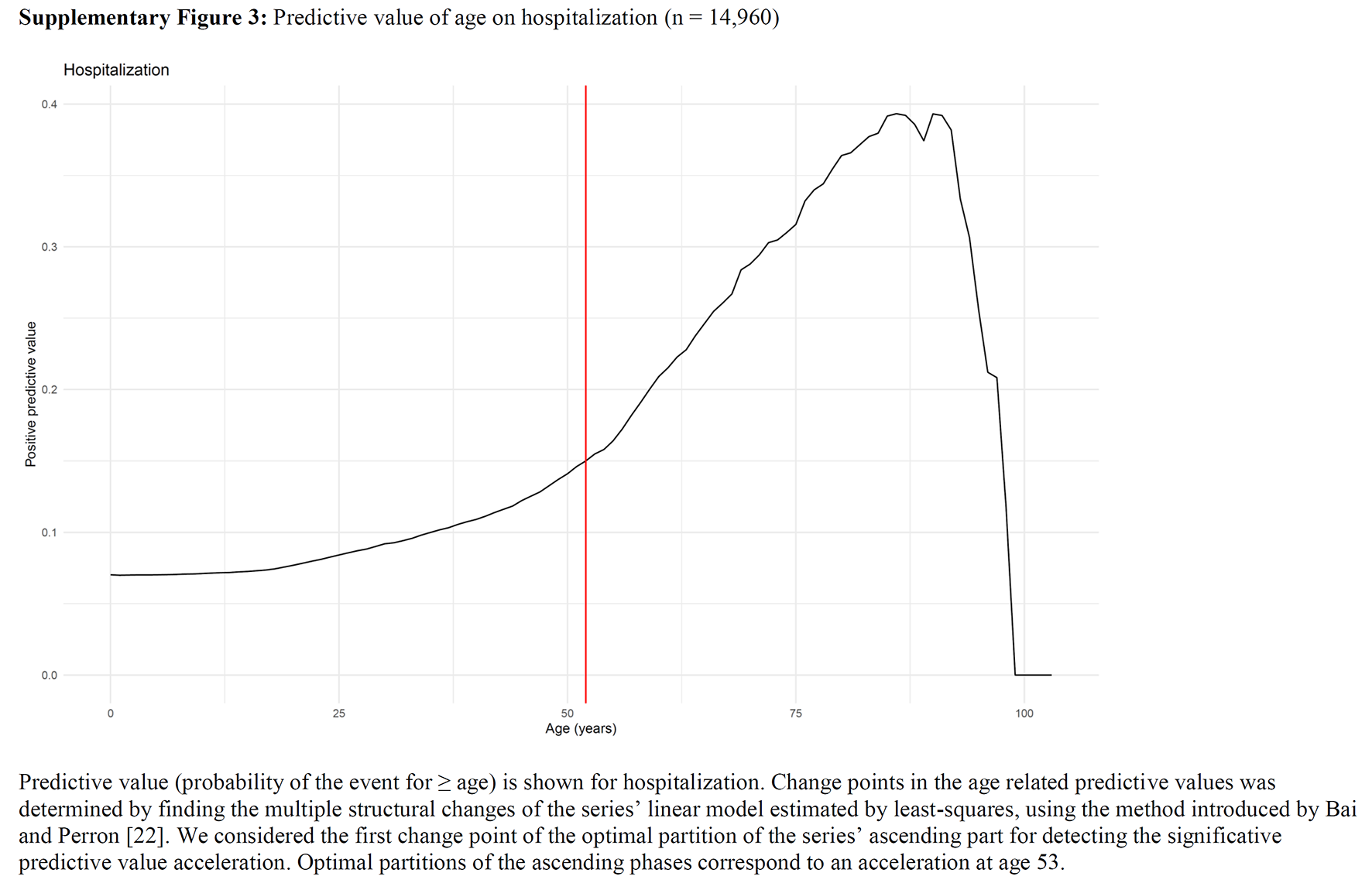

Supplement: Supplementary file 3 [file Image_3.tif]

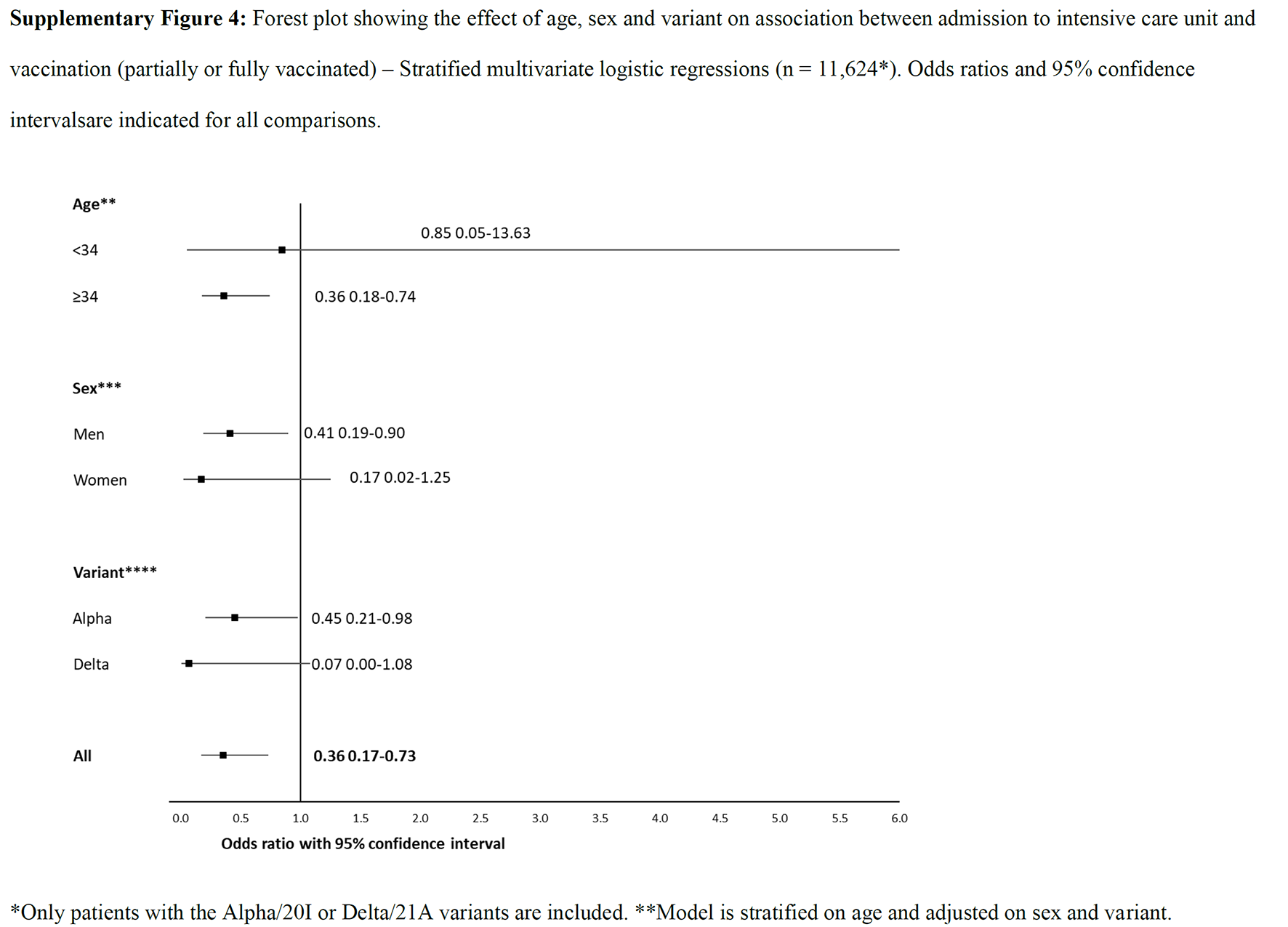

Supplement: Supplementary file 4 [file Image_4.tif]

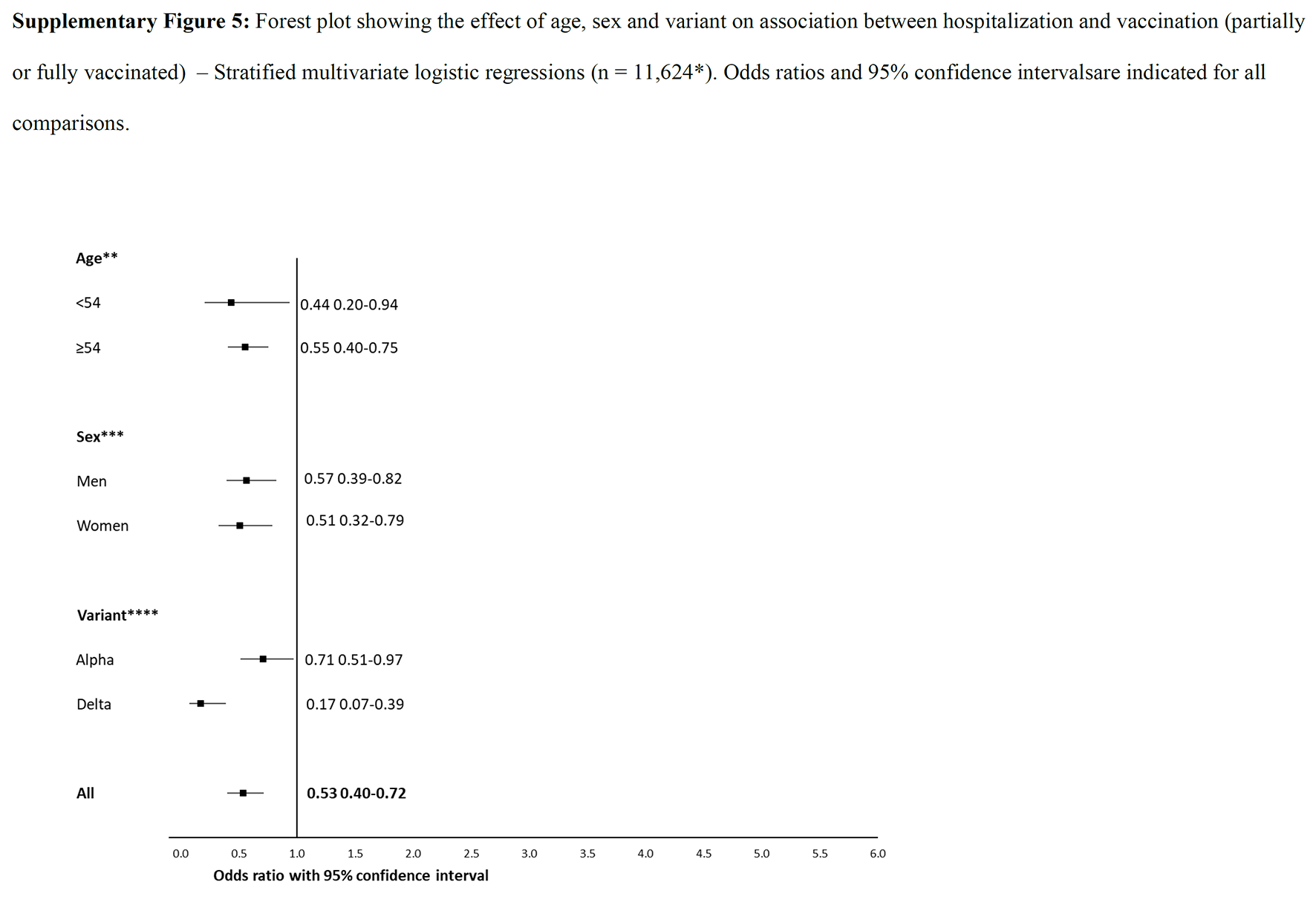

Supplement: Supplementary file 5 [file Image_5.tif]
