# Supplementary material for: SARS-CoV-2 Vaccination and Protection Against Clinical Disease: A Retrospective Study, Bouches-du-Rhône District, Southern France, 2021
Source: Front Microbiol. 2022 Jan 18;12:796807. doi: 10.3389/fmicb.2021.796807 (PMC8803903; doi:10.3389/fmicb.2021.796807)
Supplement: Supplementary file 7 [file Table_2.pdf]

Supplementary Table 2: Vaccine received by 1156* patients in the present series

**Vaccine name**	**Manufacturer**	**Number of patients***	**One/two/three doses**	**M/F**	**Age range**	**Mean age +/- SD**	**Symptomatic cases (%)**	**Hospitalization (%)**	**Admission to ICU (%)**	**Death (%)**	**Mean Ct value +/- SD**
BNT162b2	Pfizer-BioNTech	775	587/186/2	358/417	14-103	52.3 +/- 20.1	655 (84.5)	54 (7.0)	5 (0.7)	13 (1.7)	22.2 +/- 5.2
ChAdOx1-S	AstraZeneca	107	100/7	61/46	19-83	56.3 +/- 12.4	92 (86.0)	9 (8.4)	3 (2.8)	0 (0.0)	22.9 +/- 5.0
mRNA-1273	Moderna	62	56/6	32/30	19-95	43.7 +/- 20.3	53 (85.5)	2 (3.2)	0 (0.0)	0 (0.0)	21.7 +/- 5.2
Ad26.COV2.S	Johnson & Johnson	17	17/0**	9/8	29-85	51.6 +/- 17.4	16 (94.1)	1 (5.9)	0 (0.0)	0 (0.0)	19.7 +/- 5.4
ChAdOx1-S + BNT162b2	AstraZeneca/Pfizer-BioNTech	2	0/2/0	1/1	25-57	41 +/- 22.6	2 (100.0)	0 (0.0)	0 (0.0)	0 (0.0)	25.5 +/- 8.8
Spoutnik V	Gamaleya Institute	2	0/2	1/1	58-71	64.5 +/- 9.2	2 (100.0)	0 (0.0)	0 (0.0)	0 (0.0)	23.0 +/- 4.9

NA: not applicable; * the vaccine type was not available for 191 patients; **the current scheme for the the Ad26.COV2.S vaccine in France is based on a single dose.
