# Supplementary material for: SARS-CoV-2 Vaccination and Protection Against Clinical Disease: A Retrospective Study, Bouches-du-Rhône District, Southern France, 2021
Source: Front Microbiol. 2022 Jan 18;12:796807. doi: 10.3389/fmicb.2021.796807 (PMC8803903; doi:10.3389/fmicb.2021.796807)
Supplement: Supplementary file 8 [file Table_3.pdf]

Supplementary Table 3: Distribution according to SARS-CoV-2 variants of the characteristics of 1142* vaccinated patients in the present case series

Marseille IHU/WHO/Nextclade/other labels	Pangolin Lineage	Number of patients	M/F	Age range (years)	Mean age (years)	Number of symptomatic cases (%)	Number of hospitalization (%)	Number of Admission to ICU (%)	Number of death (%)	Mean Ct value **
Alpha/20I	B.1.1.7	494	237/257	18-93	58.5	406 (82.2)	50 (10.1)	7 (1.4)	7 (1.4)	22.9
Beta/20H	B.1.351	20	14/6	27-87	54.5	19 (95.0)	1 (5.0)	0 (0.0)	0 (0.0)	24.4
Delta/21A	B.1.617.2	564	269/295	14-103	41.5	459 (81.4)	6 (1.1)	0 (0.0)	3 (0.5)	22.1
Eta/21D	B.1.525	9	4/5	31-73	59.5	9 (100.0)	0 (0.0)	0 (0.0)	0 (0.0)	19.5
Gamma/20J	P.1	4	0/4	19-41	26.8	3 (75.0)	0 (0.0)	0 (0.0)	0 (0.0)	22.7
Marseille-2/20E	B.1.177	9	4/5	51-98	69.9	9 (100.0)	1 (11.1)	0 (0.0)	1 (11.1)	22.5
Marseille-4/20A.EU2	B.1.160	35	12/23	21-90	57.6	30 (85.7)	4 (11.4)	1 (2.8)	2 (5.6)	21.0
Marseille-452R/19B	A.21	2	0/2	75-91	83	2 (100.0)	1 (50.0)	0 (0.0)	0 (0.0)	21.7
Marseille-484K.V2/20B	B.1.1.318	2	1/1	40-41	40.5	2 (100.0)	0 (0.0)	0 (0.0)	0 (0.0)	22.0
Marseille-484K.V4/20A	B.1.619	1	0/1	42	NA	1 (100.0)	0 (0.0)	0 (0.0)	0 (0.0)	22.0**
Marseille-501	A.27	1	1/0	56	NA	1 (100.0)	0 (0.0)	0 (0.0)	0 (0.0)	25.8**
20A/20268G	B.1.258	1	0/1	79	NA	1 (100.0)	1 (100.0)	0 (0.0)	0 (0.0)	27.0**

F, female; M, male; NA: not applicable. * The SARS-CoV-2 genotype was not determined for 14 patients. ** Only a single value was available
