# Supplementary material for: SARS-CoV-2 Vaccination and Protection Against Clinical Disease: A Retrospective Study, Bouches-du-Rhône District, Southern France, 2021
Source: Front Microbiol. 2022 Jan 18;12:796807. doi: 10.3389/fmicb.2021.796807 (PMC8803903; doi:10.3389/fmicb.2021.796807)
Supplement: Supplementary file 9 [file Table_4.pdf]

Supplementary Table 4: Variants identified in 951* vaccinated patients according to vaccine type and doses.

**Number of doses**	**Vaccine name**	**Number of patients**	**Alpha/20I variant**	**Beta/20H variant**	**Delta/21A variant**	**Eta/21D**	**Marseille-4/20A.EU2 variant**	**Other variants**
1	BNT162b2	579	307	9	216	4	27	16
	ChAdOx1-S	99	77	6	12	1	1	2
	mRNA-1273	56	17	2	35	2	0	0
	Ad26.COV2.S	17	0	0	16	0	1	0
2¶	BNT162b2	181	61	1	112	2	3	2
	ChAdOx1-S	7	1	0	6	0	0	0
	mRNA-1273	6	0	0	6	0	0	0
	ChAdOx1-S + BNT162b2	2	0	0	2	0	0	0
	Spoutnik V	2	1	0	1	0	0	0
3¶	BNT162b2	2	0	0	2	0	0	0

* For 205 patients, informations on the vaccine type and/or variant were not available.
