# Supplementary material for: SARS-CoV-2 Vaccination and Protection Against Clinical Disease: A Retrospective Study, Bouches-du-Rhône District, Southern France, 2021
Source: Front Microbiol. 2022 Jan 18;12:796807. doi: 10.3389/fmicb.2021.796807 (PMC8803903; doi:10.3389/fmicb.2021.796807)
Supplement: Supplementary file 10 [file Table_5.pdf]

Supplementary Table 5: SARS-CoV-2 infections diagnosed at IHU Mediterranee Infection among vaccinated patients (n = 1156)

**Days after vaccination**	**Number of infected patients**
**Dose 1**	
Days 1-13	310*
Days 14 or later, before dose 2	310¶
**Dose 2**	
Days 1-13	56
Days 14 or later	148
**Dose 3**	
Days 1-13	0
Days 14 or later	2
**Unknown delay**	330

* including 7 patients vaccinated with the single dose Ad26.COV2.S vaccine; ¶including 10 patients vaccinated with the single dose Ad26.COV2.S vaccine
