# Supplementary material for: SARS-CoV-2 Vaccination and Protection Against Clinical Disease: A Retrospective Study, Bouches-du-Rhône District, Southern France, 2021
Source: Front Microbiol. 2022 Jan 18;12:796807. doi: 10.3389/fmicb.2021.796807 (PMC8803903; doi:10.3389/fmicb.2021.796807)
Supplement: Supplementary file 11 [file Table_6.pdf]

Supplementary Table 6: Comparison of studied patients according to their vaccination status and infecting SARS-CoV-2 variant (n = 14,960)

		Number	M/F	Mean age +/- SD	Median age	Symptomatic (%)	Hospitalization (%)	Admission to ICU (%)	Death (%)	Mean Ct value +/- SD
**Alpha/20I**										
	Fully vaccinated	31	21/10	69.1 +/- 14.4	74.0	24 (77.4)	5 (16.1)	0 (0.0)	2 (6.5)	22.8 +/- 4.8
	Partially vaccinated	463	216/247	57.8 +/- 15.2	59.0	382 (82.5)	45 (9.7)	7 (1.5)	5 (1.1)	22.9 +/- 4.8
	Unvaccinated	7400	3593/3807	43.5 +/- 18.8	44.0	6054 (81.8)	547 (7.4)	129 (1.7)	124 (1.7)	22.9 +/- 5.1
**Beta/20H**										
	Fully vaccinated	0	NA	NA	NA	NA	NA	NA	NA	NA
	Partially vaccinated	20	14/6	54.5 +/- 18.2	56.0	19 (95.0)	1 (5.0)	0 (0.0)	0 (0.0)	24.4 +/- 5.6
	Unvaccinated	490	247/243	42.6 +/- 17.4	43.0	400 (81.6)	38 (7.7)	10 (2.0)	5 (1.0)	22.7 +/- 5.1
**Delta/21A**										
	Fully vaccinated	121	52/69	53.2 +/-22.9	55.0	107 (88.4)	2 (1.6)	0 (0.0)	1 (0.8)	21.5 +/- 4.8
	Partially vaccinated	443	217/226	38.2 +/- 17.3	34.0	352 (79.5)	4 (0.9)	0 (0.0)	2 (0.4)	22.2 +/- 5.6
	Unvaccinated	3166	1549/1617	37.3 +/- 18.0	34.0	2524 (79.7)	126 (4.0)	28 (0.9)	23 (0.7)	21.6 +/- 5.6
**Eta/21D**										
	Fully vaccinated	1	0/1	41.0¶	NA	1(100.0)	0 (0.0)	0 (0.0)	0 (0.0)	20¶
	Partially vaccinated	8	4/4	61.9 +/- 13.1	66.5	8 (100.0)	0 (0.0)	0 (0.0)	0 (0.0)	19.2 +/- 3.2
	Unvaccinated	3	2/1	41.0 +/- 18.7	44.0	2 (66.7)	0 (0.0)	0 (0.0)	0 (0.0)	19.3 +/- 1.8
**Marseille-4/20A.EU2**										
	Fully vaccinated	2	0/2	49.5 +/- 13.4	49.5	2 (100.0)	0 (0.0)	0 (0.0)	0 (0.0)	18.4 +/- 0.5
	Partially vaccinated	33	12/21	58.1 +/- 16.4	59.0	28 (84.8)	4 (12.1)	1 (3.0)	2 (6.1)	21.0 +/- 6.3
	Unvaccinated	2454	1110/1344	48.0 +/- 20.1	48.0	1980 (80.7)	235 (9.6)	50 (2.0)	89 (3.6)	22.2 +/- 4.6
**Other**										
	Fully vaccinated	6	4/2	68.3 +/- 23.1	69.5	5 (83.3)	3 (50.0)	0 (0.0)	1 (16.7)	24.3+/-7.5
	Partially vaccinated	28	11/17	60.9 +/- 20.6	62.0	26 (92.9)	6 (21.4)	1 (3.6)	2 (7.1)	22.1 +/- 5.3
	Unvaccinated	291	144/147	45.3 +/- 20.2	44.0	225 (77.3)	34 (11.7)	12 (4.1)	28 (9.6)	22.4 +/- 5.3

¶ Only a single value was available; NA: not available.
