# Supplementary material for: SARS-CoV-2 Vaccination and Protection Against Clinical Disease: A Retrospective Study, Bouches-du-Rhône District, Southern France, 2021
Source: Front Microbiol. 2022 Jan 18;12:796807. doi: 10.3389/fmicb.2021.796807 (PMC8803903; doi:10.3389/fmicb.2021.796807)
Supplement: Supplementary file 12 [file Table_7.pdf]

Supplementary Table 7: Rate of admission to ICU according to SARS-CoV-2 variant, age and vaccinal status (n = 14,114*).

	**Variant Marseille-4/20A.EU2 (n = 2490)**					
	**Unvaccinated**			**Vaccinated (at least one dose)**		
	**n total**	**n ICU**	**ICU rate (%)**	**n total**	**n ICU**	**ICU rate (%)**
**Age**						
<34	631	6	1.0	4	0	0
≥34	1823	44	2.4	32	1	3.1
**Total**	2454	50	2.0	36	1	2.8
	**Alpha/20I variant (n = 7894)**					
**Age**						
<34	2422	8	0.3	39	0	0
≥34	4978	121	2.4	455	7	1.5
**Total**	7400	129	1.7	494	7	1.4
	**Delta/21A variant (n = 3730)**					
**Age**						
<34	1551	2	0.1	251	0	0.0
≥34	1615	26	1.6	313	0	0.0
**Total**	3166	28	0.9	564	0	0.0

*Only patients with the Alpha/20I, Delta/21A or Marseille-4/20A.EU2 variants are included.
