# Supplementary material for: SARS-CoV-2 Vaccination and Protection Against Clinical Disease: A Retrospective Study, Bouches-du-Rhône District, Southern France, 2021
Source: Front Microbiol. 2022 Jan 18;12:796807. doi: 10.3389/fmicb.2021.796807 (PMC8803903; doi:10.3389/fmicb.2021.796807)
Supplement: Supplementary file 13 [file Table_8.pdf]

Supplementary Table 8: Rate of hospitalization according to SARS-CoV-2 variant, age and vaccinal status (n = 14,114*).

	**Marseille-4/20A.EU2 Variant (n = 2490)**					
	**Unvaccinated**			**Vaccinated (at least one dose)**		
	**n total**	**n Hospitalization**	**Hospitalization rate (%)**	**n total**	**n Hospitalization**	**Hospitalization rate (%)**
**Age**						
<54	1489	54	3.6	12	1	8.3
≥54	965	181	18.8	24	3	12.5
**Total**	2454	235	9.6	36	4	11.1
	**Alpha/20I variant (n = 7894)**					
**Age**						
<54	5057	163	3.2	159	6	3.8
≥54	2343	384	16.4	335	44	13.1
**Total**	7400	547	7.4	494	50	10.1
	**Delta/21A variant (n = 3730)**					
**Age**						
<54	2544	59	2.3	403	0	0
≥54	622	67	10.8	161	6	3.7
**Total**	3166	126	4.0	564	6	1.1

*Only patients with the Alpha/20I, Delta/21A or Marseille-4/20A.EU2 variants are included.
